# Ultra-Broadband Polarization Conversion Metasurface with High Transmission for Efficient Multi-Functional Wavefront Manipulation in the Terahertz Range

**DOI:** 10.3390/nano11112895

**Published:** 2021-10-29

**Authors:** Xiaoqiang Jiang, Wenhui Fan, Chong Qin, Xu Chen

**Affiliations:** 1 State Key Laboratory of Transient Optics and Photonics, Xi’an Institute of Optics and Precision Mechanics, Chinese Academy of Sciences, Xi’an 710119, China; jiangxiaoqiang@opt.cn (X.J.); qinchong@opt.cn (C.Q.); chenxu@opt.ac.cn (X.C.); 2 Center of Materials Science and Optoelectronics Engineering, University of Chinese Academy of Sciences, Beijing 100049, China; 3 Collaborative Innovation Center of Extreme Optics, Shanxi University, Taiyuan 030006, China

**Keywords:** terahertz, metasurface, polarization conversion, wavefront manipulation, mode purity

## Abstract

Recently, terahertz (THz) wireless communication has been widely investigated as the future prospect of wireless network architecture. However, most of the natural existing materials are inapplicable for THz devices, which hinder their further development. To promote the integration and channel capacity of the THz wireless communication systems, an ultrabroadband polarization conversion metasurface for efficient multi-functional wavefront manipulation is proposed. The designed metasurface is composed of an arrow-type structure sandwiched by a pair of orthogonal gratings, which can induce the Fabry-Pérot-like cavity for improving the transmission. Simulated results indicate that the transmission coefficient of the cross-polarization metasurface is higher than 90% from 0.73 THz to 2.24 THz, and the corresponding polarization conversion ratio is greater than 99.5%. Moreover, the phase coverage of 0–2π at operation frequency can be easily obtained by altering the geometric parameter of the metasurface. To demonstrate the concept of wavefront manipulation, anomalous refraction, focusing metalens, and vortex beam generation are investigated in detail. All of these applications exhibit a remarkable performance of the proposed metasurface that has great potential in prompting the efficient, broadband and compact systems for THz wireless communication.

## 1. Introduction

The desired manipulation of the electromagnetic (EM) wave, which exhibits a magnificent prospect for next-generation applications, has gradually consolidated its position as motivating research in past years [1,2,3]. However, the permittivity of natural materials is only within a limited range that hinders their capability to control the EM wave. Moreover, the phase accumulation of conventional EM devices usually requires bulk size and certain geometrical shapes to provide the adequate propagation distance, which inevitably introduce inconvenience and inefficiency for practical utilizations [4]. Fortunately, the emergence of metasurfaces provides a much better opportunity to realize the manipulation of a transmitted or reflected EM wave in desired ways [5]. Metasurface as an ultrathin surface, also considered as two-dimensional (2D) metamaterial [6,7], is composed of planar meta-atoms following certain orders to accomplish preset EM responses. Instead of accumulating the phase in free space, metasurfaces control the EM wave by appropriately arranging the meta-atoms to exploit the abrupt phase discontinuities between their adjacent elements [8]. Therefore, the thickness of metasurface can be much thinner than the operation wavelength, which contributes to the alleviation of intrinsic losses from material, especially for metallic structures. The advantages make metasurface an ideal candidate when the compact size, multi-functional effects, high efficiency and broadband operation are required for beam shaping. Yu et al. firstly proposed a generalized version of Snell’s law and the anomalous light propagation was realized by changing the angle of V-shape metasurfaces to cover the 2π phase gradient [9]. Since then, a variety of metasurfaces based on phase gradient were proposed with different functional features such as polarization convertors [10,11,12], anomalous propagation [13,14], focusing lens [15,16], vortex beam generation [17,18,19], holograms [20,21] and cloaking [22].

Recently, terahertz (THz) wireless communication has been widely investigated as the future prospect of wireless network architecture due to the urgent demands of communication speed and capacity [23,24]. Since most of the natural existing materials are inapplicable for THz devices, the metasurfaces for efficient wavefront manipulation have drawn great attention in developing THz wireless communication systems based on two main reasons: (1) the compact size of metasurfaces can greatly miniaturize the functional devices and (2) the vortex beam, one of the most important applications of metasurfaces, can infinitely increase the channel capacity of wireless communication systems in theory [14,18,25]. The previous researches of metasurfaces have primarily focused on reflection-type structures, since high reflectance can be easily obtained by adding a metallic plate under the structure [26,27,28]. However, the development of transmission-type metasurfaces, which are more suitable for practical applications, have been severely restricted owing to insufficient transmission. The single-layer metallic structures are impossible to realize 2π phase delay accompanied with high transmission, and their cross-polarization transmission have been proven to be less than 50% [29]. In order to solve the defects, the metasurfaces with multi-layer structure have been proposed to efficiently manipulate the wavefront of transmitted waves [30,31]. Among the structures, tri-layered metasurfaces are the best selection for efficient wavefront manipulation. By independently designing each layer, the high cross-polarization transmission along with 2π phase delay can be achieved without any restriction from the EM boundary conditions [32]. Grady et al. designed a polarization conversion metamaterial with tri-layer structure for achieving anomalous refraction in the THz range [10]. Yang et al. experimentally investigated flat lens composed of three broadband metasurfaces for efficient THz wave control [30]. Liu et al. presented coding metasurfaces with three metallic layers to bend THz beams and generate Bessel-Beam [32]. Fan et al. designed a tri-layered metasurface for wavefront manipulation in the THz range [33]. Although remarkable progress has been accomplished, the metasurface with high transmission and broadband characteristic is still a great challenge that needs to be further investigated.

In this article, a tri-layered metasurface composed of an arrow-type structure sandwiched by a pair of orthogonal gratings is proposed and investigated in detail. The ultra-broadband polarization conversion metasurface can realize multi-functional wavefront manipulation with remarkable performance in the THz range. The transmission coefficient of the polarization conversion metasurface is higher than 90% from 0.73 THz to 2.24 THz and the corresponding polarization conversion ratio (PCR) is greater than 99.5%. In addition, the phase coverage of 0–2π at operation frequency can be easily obtained in the proposed metasurface by altering the geometric parameter. As the concept demonstration, the anomalous refraction, cylindrical focusing, point focusing and vortex beams with varied topological charge are investigated. The simulated results indicate that the anomalous refraction can be realized from 1.0 THz to 1.9 THz, and the focusing efficiency of cylindrical and point focusing metalens are 69.9% and 82.4%, respectively. Moreover, the mode purity is also considered to evaluate the efficiency of vortex beams and all the vortex beams have high mode purity greater than 90%. In addition, the broadband operation of point focusing and vortex beam is also investigated, which exhibits stable performances in a certain frequency range. The remarkable results show the proposed metasurface has tremendous potential in prompting the efficient, broadband and compact systems for THz wireless communication.

## 2. Structural Design and Cross-Polarization Conversion

**Figure 1:**
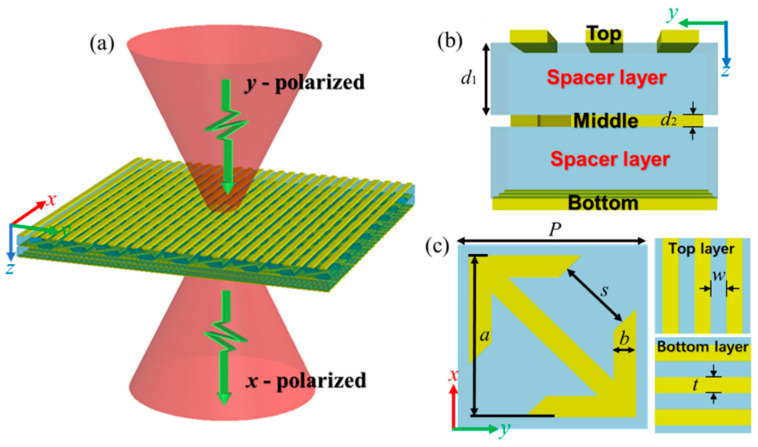
(**a**) Illustration of the polarization conversion metasurface. (**b**) The side view of the unit cell at *y*-*z* plane. (**c**) The view of the layer at *x*-*y* plane.

First of all, the unit-cell of the metasurface is designed to perform a selection of linear polarization with a controlled phase delay. Figure 1a conceptually illustrates the proposed metasurface for cross-polarization conversion: when incident THz waves with *y*-polarization cross the metasurface, it can be converted into *x*-polarized transmitted waves. The details of the proposed metasurface are shown in Figure 1b,c. The tri-layered metasurface consists of three independent metallic structures isolated by spacer layers. To be specific, the arrow-type metallic structure in the middle layer is expected to realize high transmission of polarization conversion and 2π phase delay. The orthogonal gratings at the top and bottom layers are mainly responsible for selecting the polarization of incident waves. The optimized geometrical parameters are: *P* = 60 μm, *s* = 24.5 μm, *a* = 52.5 μm, *b* = 7 μm, *w* = 10 μm, *t* = 10 μm, *d*_1_ = 17 μm and *d*_1_ = 3 μm.

By employing the electromagnetic simulation based on the finite integrated method, the proposed metasurface and its applications are numerically investigated. The incident waves are *y*-polarized plane waves along +*z* direction, and the periodical boundary is applied in *x*- and *y*-axis. Moreover, the input material parameters are taken from the material library. Specifically, the aluminum is selected to comprise of the metallic structure with conductivity *σ* = 3.56 × 10^7^ S/m as well as the polyimide for the spacer layer with relative permittivity *ε_r_* = 3.5 and loss tangent of 2.7 × 10^−3^ due to its high transparency in THz range [34].

**Figure 2:**
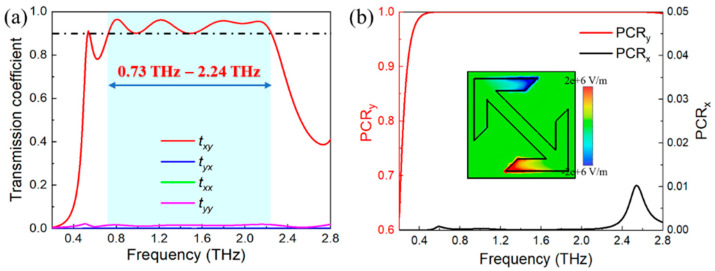
(**a**) The transmission coefficients of proposed metasurface. (**b**) The corresponding polarization conversion and the insertion is electric field distribution of middle layer at 1 THz.

The cross-polarization conversion is firstly investigated in this section. From Figure 1c, the proposed metasurface can be divided into three functional parts: top (perpendicular to the incident waves), middle (arrow-type structure) and bottom (parallel to the incident waves), which are separated by the spacer layer. The top and bottom layers are employed as polarization selectors, while the middle layer performs the polarization convertor. To be specific, incident waves with *y*-polarization can cross the perpendicular top gratings, but they are blocked by the parallel bottom gratings. Meanwhile, only the *x*-polarized waves are able to pass through the bottom gratings as transmitted waves in the same way. During the conversion, the electric dipole is excited from the middle layer, as depicted in the insertion of Figure 2b, which can be coupled into a certain component of incident waves to realize the conversion of linear cross-polarization [35]. The remarkable transmission of cross-polarization is ascribed to the resonance of Fabry-Pérot-like cavity, which causes repeated reflections and transmissions inside the tri-layered structure [36].

The responses of the proposed metasurface to incident waves with *y*-polarization are shown in Figure 2a. The transmission coefficients of co-polarization waves are fully identical (*t_xx_* = *t_yy_*, green and purple curves) and they are all restricted to 2% from 0.2 THz to 2.8 THz. On the contrary, the cross-polarized waves *t_xy_* (*y*-polarization to *x*-polarization, red curve) and *t_yx_* (*x*-polarization to *y*-polarization, blue curve) have distinctly different transmission coefficients. The *t_xy_* is greater than 90% between 0.73 THz and 2.24 THz (blue area), implying that the polarization of incident waves has almost been converted into the cross-polarized transmitted waves. Simultaneously, the *t_yx_* is strongly suppressed to be less than 0.1% in the whole operation frequency. It is also worth pointing out that when the incident waves with *x*-polarization cross the metasurface along −*z* direction, the *y*-polarized transmitted waves with completely identical performances can be obtained. Therefore, the proposed metasurface can be functional in both +*z* and −*z* directions.

Moreover, the PCR is also investigated to further characterize the cross-polarization of the proposed metasurface by calculating the following equations [33]:

(1) $${\mathrm{PCR}}_{x}=\frac{{\left|t_{yx}\right|}^{2}}{{\left|t_{xx}\right|}^{2}+{\left|t_{yx}\right|}^{2}},{PCR}_{y}=\frac{{\left|t_{xy}\right|}^{2}}{{\left|t_{yy}\right|}^{2}+{\left|t_{xy}\right|}^{2}}$$

From Figure 2b, the PCR*_y_* (*y*-polarization to *x*-polarization) is higher than 99.5% and the PCR*_x_* (*x*-polarization to *y*-polarization) is limited to 0.01% between 0.73 THz and 2.24 THz. Moreover, the corresponding relative bandwidth can be obtained from BF = 2 × (*f_h_* − *f_l_*)/(*f_h_* + *f_l_*), where *f_h_* and *f_l_* are two cutoff frequencies [37]. Accordingly, the calculated BF is up to 101.7% (here, *f_h_* = 2.24 THz and *f_l_* = 0.73 THz), which is better than that of previous reports [10,12,33,37]. The preferable results indicate that the dissipation from the material losses can be neglected. Therefore, the metasurface is able to become a broadband polarization convertor with remarkable conversion efficiency.

**Figure 3:**
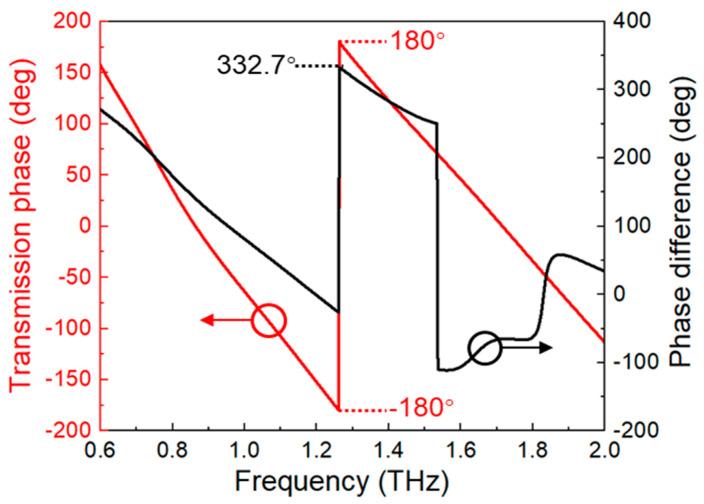
The transmission phase (red curve) and phase difference (black curve) of the metasurface.

The transmission phase of the proposed metasurface is also investigated. In Figure 3, the 2π phase delay can be achieved around 1.27 THz (red curve). In addition, the phase difference is also considered to illustrate the mechanism of the phase delay caused by the designed metasurface [38]. The phase difference *φ_d_* can be obtained from *φ_d_* = *φ_m_* − *φ_f_*, where *φ_m_* and *φ_f_* are referring to the transmission phase in the metasurface and free space, respectively. The simulated results show that the phase difference is 332.7° (black curve), implying that the metasurface has an excellent capability for phase modulation.

## 3. Multi-Functional Wavefront Manipulation

In this section, the phase gradient is introduced into the metasurface to investigate the applications of wavefront manipulation. By altering the geometrical parameter *s* (Figure 1c), the complete phase coverage of 0–2π at operation frequency can be attained, which is considerably crucial for discretionary wavefront manipulation. As the concept demonstration, anomalous refraction, cylindrical focusing, point focusing, and vortex beam generation are investigated in detail, respectively. It is essential to point out that only the middle layer of these applications is depicted as the schematic for clarity.

The same computation of electromagnetic is also used in this section, but with different boundary conditions. To be specific, open boundary condition is applied in *x*-, *y*- and *z*-axis. The plane wave with *y*-polarization is defined in the simulation of anomalous refraction, cylindrical focusing, and point focusing. The *y*-polarized Gaussian beam is utilized as the excitation for the vortex beam generator in order to avoid the truncation effect induced by the metasurface edges [33]. Moreover, the electric field monitor and far-field monitor at operation frequency are also employed to observe the simulated results. At least 64 GB of computer memory is required due to the relatively high accuracy of the calculation in the time-domain solver.

### 3.1. Anomalous Refraction

**Figure 4:**
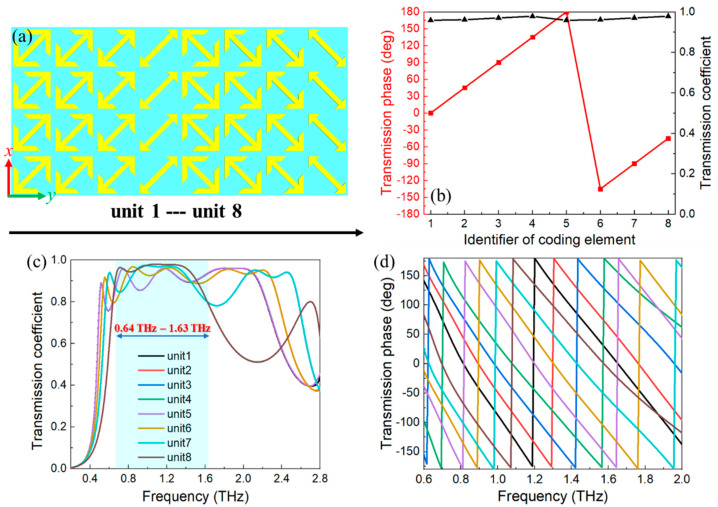
(**a**) The schematic of designed anomalous refractor (only the middle layer is depicted for clarity). (**b**) The transmission coefficients and phase of cross-polarized THz wave for units 1 to 8 at 1.2 THz. (**c**,**d**) The transmission coefficients and phase delay of cross-polarized THz wave for units 1 to 8, respectively.

The anomalous refraction based on the proposed metasurface is investigated in this section. Specifically, the normal incident waves with *y*-polarization can be transformed into *x*-polarized transmitted waves with anomalous refraction. Primarily, because the required phase compensations can be easily satisfied by altering the parameter *s* of the metasurface, the cross-polarization transmission *t_xy_* is a priority to be considered. To realize the anomalous refraction of transmitted waves, a supercell consisting of eight elements was assembled in this section. The elements with various parameters and orientations were selected to satisfy the required phase compensations accompanied with high transmission and marked as unit 1 to unit 8 in Figure 4a. The arrow orientations of units 1 to 4 were the same and the parameters *s* were 19.8 μm, 27.3 μm, 35.3 μm and 52.6 μm, respectively. Meanwhile, units 5 to 8 were attained by taking the mirror symmetry of the aforementioned four elements. Therefore, they share fully identical transmission but the additional phase delay of π was introduced into units 5 to 8. Figure 4b depicts the phase shift and transmission coefficient of units 1 to 8 at the preset frequency of 1.2 THz, where the supercell has a phase gradient by step of π/4 and the transmission coefficients of all elements are higher than 95%. Moreover, the transmission coefficients of all elements are greater than 80% with 2π phase delay between 0.64 THz and 1.63 THz, as depicted in Figure 4c,d.

The phenomenon of anomalous refraction can be achieved by the assembled supercell based on the generalized version of Snell’s law [9]:

(2) $$\sin(\theta_{t})n_{t}-\sin(\theta_{i})n_{i}=\frac{\lambda_{0}}{2\pi }\frac{d\varphi }{dx}$$

where *θ_t_* and *θ_i_* denotes the refraction angle and the incident angle, respectively, *n_t_* and *n_i_* are the refractive indices of the surrounding medium (air here), *λ*_0_ represents the wavelength of incident waves and *dφ*/*dx* is the phase gradient. Here, the supercell is in free space and the excitation is a normal incident plane wave, implying that *n_t_* = *n_i_* = 1 and *θ_i_* = 0. Eventually, the *θ_t_* can be theoretically calculated by the simplified equation:

(3) $$\theta_{t}=\arcsin(\lambda_{0}/pN)$$

where *p* represents the period of unit cell and *N* denotes the total number of elements within the assembled supercell. Therefore, the anomalous refraction angle *θ_t_* at operation frequency can be theoretically obtained by Equation (3) to compare with the following simulation results.

**Figure 5:**
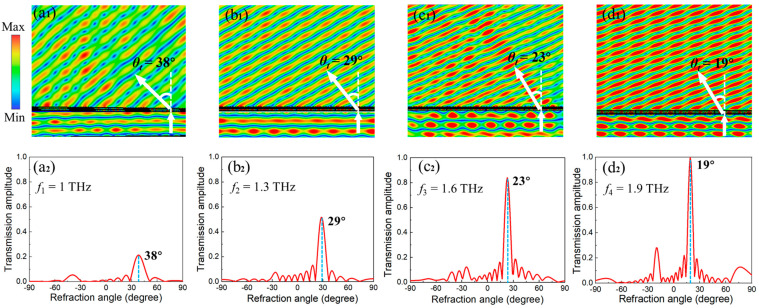
(**a_1_**–**d_1_**) The electrical field in *x*-*z* plane. (**a_2_**–**d_2_**) The normalized far-field power intensity in *x*-*z* cutting plane at four typical frequencies, respectively.

To intuitively demonstrate the anomalous refraction, the electrical field of the designed supercell in the *x*-*z* plane was simulated at a frequency between 1 THz to 1.9 THz by step of 0.3 THz, as depicted in Figure 5(a_1_–d_1_). The supercell is illuminated by a normal incident THz wave with *y*-polarization. It is clear that the wavefront of transmitted waves is clearly deflected in an anomalous direction, indicating that the incident waves have been converted into *x*-polarized transmitted waves propagating along the anomalous direction. The normalized far-field power intensity along the *x*-*z* plane was also calculated to show the corresponding refraction angle *θ_t_*, which were 38° at 1 THz, 29° at 1.3 THz, 23° at 1.6 THz and 19° at 1.9 THz from Figure 5(a_2_–d_2_), respectively. Meanwhile, the theoretical predictions from Equation (3) are 38.65° at 1 THz, 28.74° at 1.3 THz, 22.98° at 1.6 THz and 19.19° at 1.9 THz, respectively. The results indicate that the simulations agree well with the theoretical predictions, implying that the designed metasurface can function as a broadband anomalous refractor with excellent performance.

### 3.2. Focusing Metalens

The focusing lens is one of the most important devices in optical applications since they are indispensable components in practically all complex systems, including imaging and wireless communication systems. Conventional focusing lenses inevitably have certain curvatures and bulk size for accumulating sufficient phase compensations, which severely hinder their integrated-optics applications. Fortunately, the development of metalens provides an excellent platform to simplify the optical components that notably prompt the integration of optical systems. Here, two kinds of metalens including cylindrical focusing and point focusing have been accomplished by delicately arranging the phase gradient metasurfaces. In this case, the definition of focusing efficiency is the ratio of integrated power within the area having radius of 2 × FWHM (full width at half maximum) to the power of incident waves [39].

**Figure 6:**
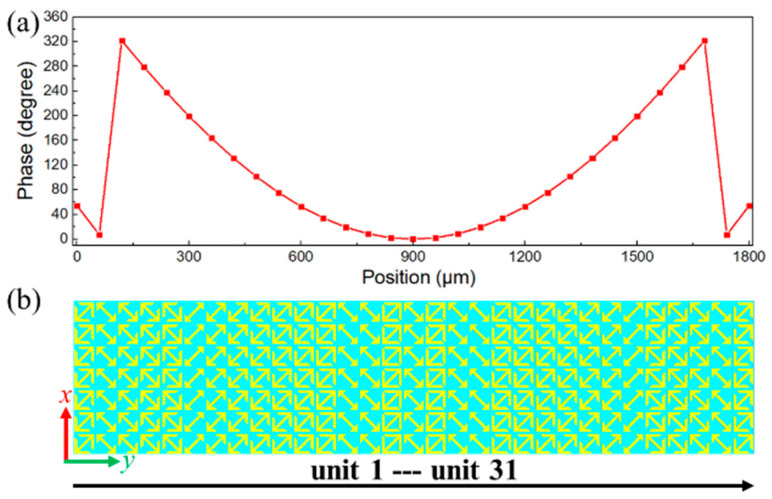
(**a**) The required phase compensations by step of 60 μm. (**b**) The middle layer of the 1D metalens in *x*-*y* plane (only the middle layer is depicted for clarity).

To begin with, a one-dimensional (1D) metalens with an efficient cylindrical focus working at 1 THz was designed by arranging the metasurface units. When the incident waves with *y*-polarization transmit along +*z* direction, the required phase compensations can be accumulated on the propagation path, and eventually converted into the *x*-polarized spherical waves. The phase compensations at *x*-axis are expressed as [33]:

(4) $$\varphi (x,f)=\frac{2\pi }{\lambda }(\sqrt{x^{2}+f^{2}}-f)$$

where *λ* denotes the working wavelength and *f* represents the focal length, which are set as 300 μm (1 THz) and 1000 μm, respectively. From Equation (4), the required phase compensations at *x*-axis by step of 60 μm (periodical length of the unit cell) are theocratically obtained and depicted in Figure 6a. Similarly, the transmission coefficient is also a priority in this case. The transmission coefficients of selected elements are higher than 88.5% and the corresponding PCR*_y_* is higher than 96%, which can contribute to the tempting focusing efficiency of designed metalens. The middle layer of proposed cylindrical metalens is shown in Figure 6b, which has 31 elements with varied orientations and parameters along *x*-axis.

**Figure 7:**
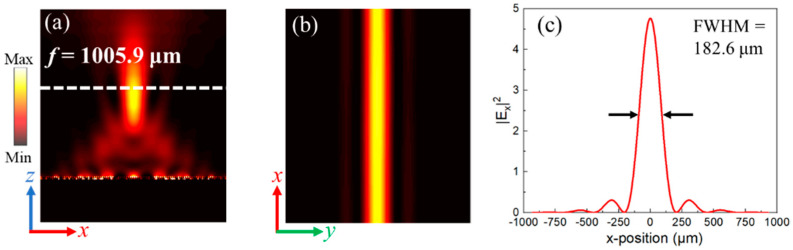
The electrical field distributions of cross-polarized waves |*E_x_*|^2^ in (**a**) *x*-*z* plane and (**b**) *x*-*y* plane. (**c**) The profile of |*E_x_*|^2^ in focal plane.

The incident waves are the *y*-polarized plane wave along +*z* axis with frequency of 1 THz and the total length of the simulation area in *z*-direction is 2500 μm. The electrical field of the *x*-polarized waves was calculated to illustrate the phenomenon of cylindrical focusing. From Figure 7a, the strongest focus is obtained at *f* = 1005.9 μm, which is similar to the preset focal length (*f* = 1000 μm). Figure 7b intuitively demonstrates that the transmitted waves with *x*-polarization have been focused into a column at the center of *x*-*y* plane. The numerical results in Figure 7c demonstrate that the FWHM of spherical waves is 182.6 μm. Moreover, the maximum value of the electrical field at the focal center is 4.7 times stronger than that of the incident waves and the focusing efficiency of designed 1D metalens is 69.9%, implying the remarkable performance of cylindrical focusing.

In addition, the 2D metalens with point focusing was also investigated by arranging the metasurface units at *x-y* plane. When the incident waves with *y*-polarization transmit along +*z* direction, the required phase compensations can be accumulated on the propagation path. Eventually, the transmitted waves with *x*-polarization focused into a point at *x*-*y* plane can be obtained. In order to realize point focusing, the 2D metalens need to satisfy the certain order of the phase compensations which are defined as [14]:

(5) $$\varphi (x,y,f)=\frac{2\pi }{\lambda }(\sqrt{x^{2}+y^{2}+f^{2}}-f)$$

**Figure 8:**
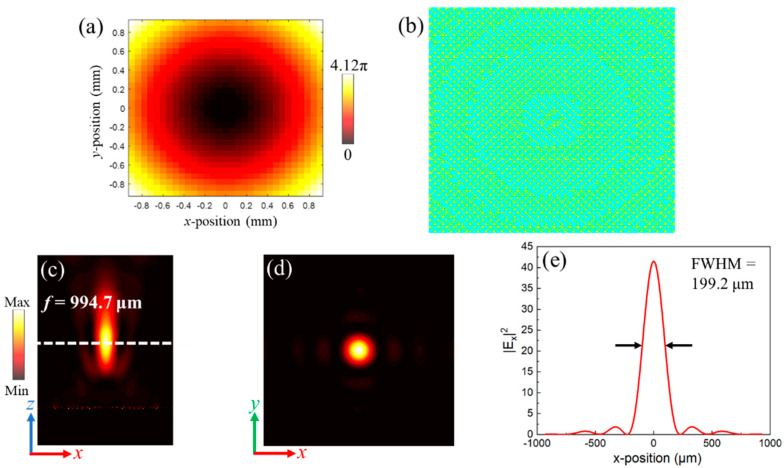
(**a**) The required phase compensations by step of 60 μm in *x*-*y* plane. (**b**) The middle layer of designed 2D metalens (only the middle layer is depicted for clarity). The electrical field of cross-polarized waves |*E_x_*|^2^ in (**c**) *x*-*z* plane and (**d**) *x*-*y* plane. (**e**) The profile of |*E_x_*|^2^ in the focal plane.

In this case, the working wavelength was set as 300 μm (1 THz) and the preset focal length was *f* = 1000 μm. From Equation (5), the required phase compensations at *x*-*y* plane by step of 60 μm along both *x*- and *y*-axis are theoretically obtained and shown in Figure 8a. Meanwhile, the middle layer of the proposed 2D metalens is shown in Figure 8b, composed of 31 × 31 elements. The transmission coefficients of the elements are higher than 88.5% with corresponding PCR*_y_* higher than 96%, which can substantially improve the efficiency of the designed 2D metalens. Moreover, the electrical field of *x*-polarized waves is calculated to illustrate the phenomenon of point focusing. From Figure 8c, the practical focal length in the simulation is *f* = 994.7 μm, which is approximate to the preset focal length (*f* = 1000 μm). Figure 8d denotes that the point focusing can be generated from the designed 2D metalens on the focal plane. The simulated results demonstrate that the FWHM is 197.2 μm at the focal center, as depicted in Figure 8e. Moreover, the maximum value of electric field at the focal center is 41.8 times stronger than that of incident waves, and focusing efficiency of the designed 2D metalens is 82.4%, implying the significant performance of the point focusing.

**Figure 9:**
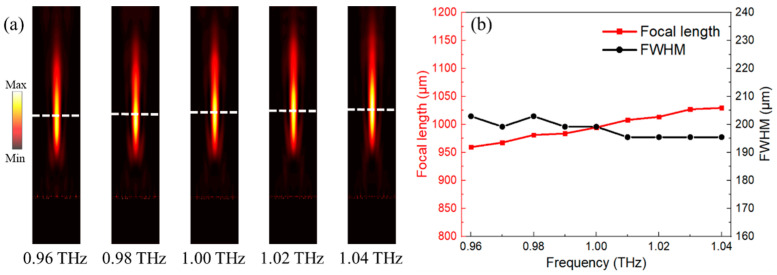
(**a**) The electrical field of cross-polarized waves in *x*-*z* plane from 0.96 THz to 1.04 THz. (**b**) The focal length and FWHM of the point focusing from 0.96 THz to 1.04 THz.

It is worth noting that the frequency of incident waves may be shifted in a practical application due to the varied working conditions. Therefore, the performances of the designed point focusing metalens with operation frequency ranging from 0.96 THz to 1.04 THz have been simulated to demonstrate the broadband operation. From Figure 9a, the electrical field of the cross-polarized waves in *x*-*z* plane indicates that the focal point barely changes between 0.96 THz and 1.04 THz. Moreover, the focal length and FWHM of the focal point at different frequency are also considered and depicted in Figure 9b. As the working frequency rises from 0.96 THz to 1.04 THz, the focal length (red) of the 2D metalens almost linearly increases from 959.6 μm to 1029.7 μm. Moreover, the corresponding FWHM (black) at the focal plane ranges from 195.4 μm to 202.9 μm. The slight variations indicate the great stability of the 2D metalens in broadband operation.

### 3.3. Vortex Beam Generation

The vortex beam accompanied with orbital angular momentum (OAM), which exhibits the helical phase distribution, is of great interest in the THz range to satisfy the demand of sixth-generation (6G) wireless communication presented by the International Telecommunication Union [40]. The OAM can infinitely increase the capacity of wireless communication system in theory, because it has countless and mutually orthogonal eigenstates [18]. By appropriately arranging the metasurface units, the vortex beams at various OAM modes (topological charge *l* = ±1 and ±2) are demonstrated in this section. To be specific, when the *y*-polarized incident waves transmit along +*z* direction to accumulate the required phase compensations on the propagation path, the transmitted vortex beams with *x*-polarization can be eventually generated. Moreover, the mode purity is also considered to evaluate the efficiency of the vortex beams.

In order to realize vortex beam generation, the required phase compensations of the proposed metasurfaces at *x*-*y* plane need to be properly arranged to satisfy the certain phase compensations, which are defined as [18]:

(6) $$\varphi_{l}(x,y)=l\times \arctan(y/x)$$

**Figure 10:**
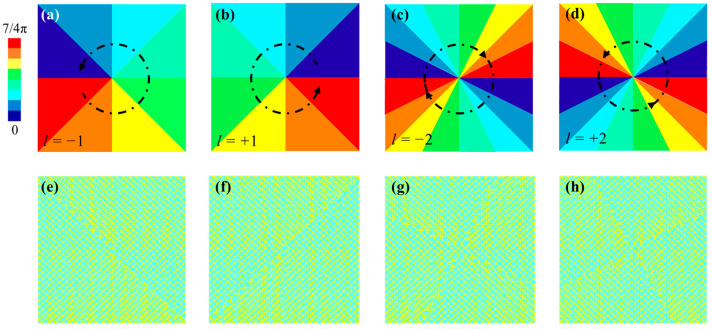
The required phase compensations for vortex beam generators (**a**) *l* = −1, (**b**) *l* = +1, (**c**) *l* = −2, (**d**) *l* = +2. (**e**–**h**) are corresponding vortex beam generators (only the middle layer is depicted for clarity).

In this case, the proposed vortex beam generators are equally divided into 8 and 16 regions referring to *l* = ±1 and ±2, respectively. Therefore, each triangular region is needed to satisfy the certain phase compensations defined as:

(7) $$\varphi_{l}(x,y)=\frac{2\pi }{N}\left[\frac{l\cdot \arctan(y/x)}{2\pi /N}+1\right]$$

where *N* denotes the total number of triangular regions. Therefore, the required phase compensations are calculated and depicted in Figure 10a–d for *l* = ±1 and ±2, respectively, where the phase gradient of π/4 is maintained in the adjacent triangular regions. For clarity, the increase in the demanded phase compensations along the azimuthal angle is represented by the black arrow shown in Figure 10a–d. The corresponding vortex beam generators with 22 × 24 elements are shown in Figure 10e–h.

**Figure 11:**
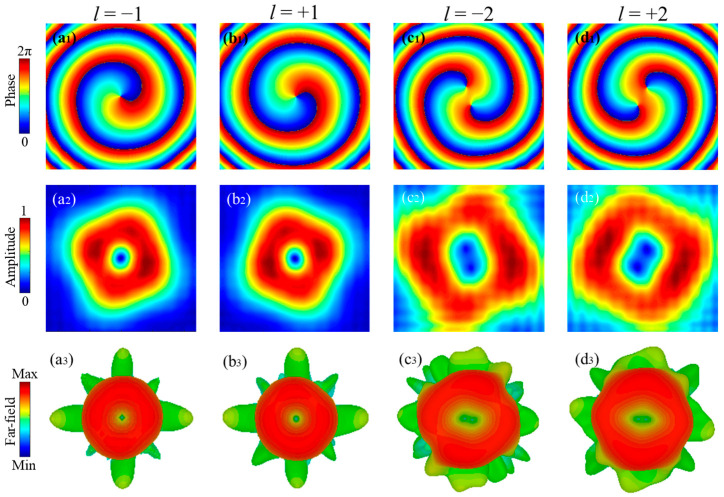
The phase, amplitude and far-field profile in *x*-*y* plane at 1 THz with (**a**) *l* = −1, (**b**) *l* = +1, (**c**) *l* = −2, (**d**) *l* = +2, respectively.

The phase, amplitude and far-field profile of the vortex beams working at 1 THz generated from the vortex beam generators are depicted in Figure 11. From Figure 11(a_1_,a_2_), it is clear that the *x*-polarized vortex beam with *l* = −1 is achieved from the generator with eight adjacent regions (Figure 10a) illuminated by *y*-polarized incident waves. The vortex beam with *l* = −1 carriers a spiral phase and have a null-amplitude area at the center, which is further verified by the far-field profile in Figure 11(a_3_). Similarly, those features also exist in the vortex beam with *l* = +1 except the opposite phase rotation of spiral arms, and the corresponding phase, amplitude and far-field profile in *x*-*y* plane at 1 THz are also depicted in Figure 11(b_1_–b_3_). The inversed spiral arms are in consequence of the opposite topological charge, which requires the symmetric phase compensations. Moreover, the metasurfaces with sixteen adjacent regions, depicted in Figure 10c,d, are able to generate the vortex beams with *l* = −2 and +2 at 1 THz in the far-field, as shown in Figure 11(c_3_,d_3_), respectively. Similarly, they both exhibit two null-amplitude area at the center region, but their spiral phase rotations are in opposite directions, as shown in Figure 11(c_2_,d_2_) and (c_1_,d_1_), respectively.

To identify the performance of the designed vortex beam generators, the mode purity has been investigated using the Fourier transform [41]:

(8) $$\alpha (\varphi )=\sum_{-\infty }^{+\infty }(\frac{1}{2\pi }\int_{-\pi }^{\pi }\alpha (\varphi )e^{-jl\phi }d\varphi )\times e^{jl\varphi }$$

where *α*(*φ*) is the phase sample and *e^−jlφ^* is the spiral harmonics.

**Figure 12:**
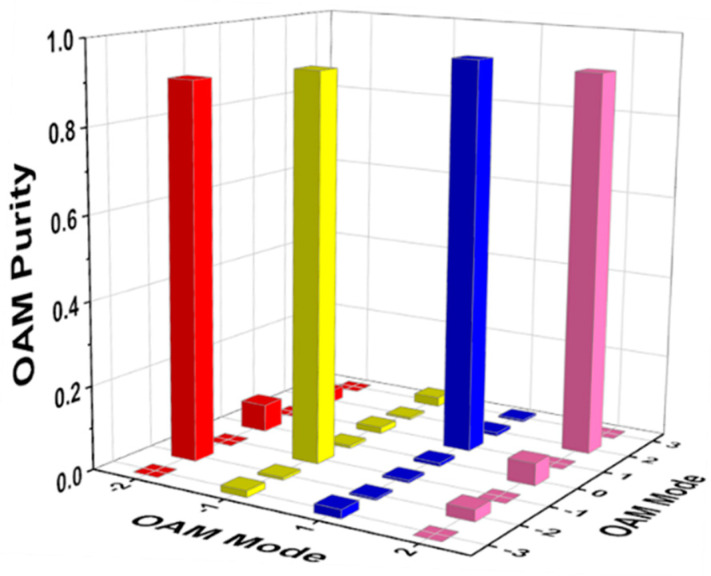
The OAM purity of vortex beams at 1 THz.

The calculated OAM purity is depicted in Figure 12. It is clear that the mode purity of the vortex beam with *l* = ±1 is up to 92.8% (yellow) and 94.5% (blue) at 1 THz, respectively. When the vortex beam with *l* = ±2 is located at 1 THz, the mode purity is up to 90.1% (red) and 91.8% (pink), respectively. It can also be observed that some parasitic phase noises are also generated at other modes, but they can be negligible compared to the operation mode. In general, all the designed vortex generators exhibit excellent performance with extremely high mode purity greater than 90%.

**Figure 13:**
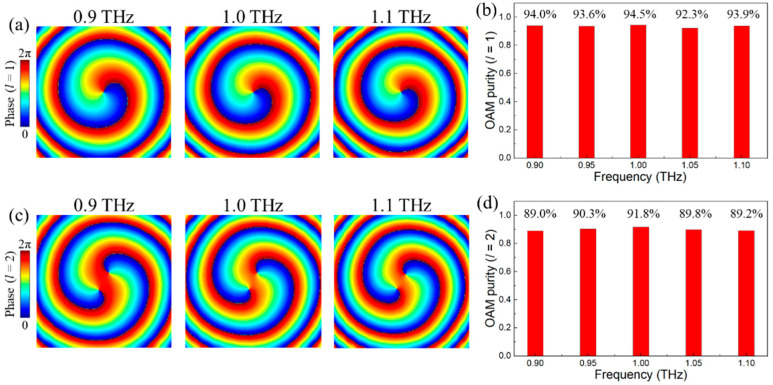
(**a**) and (**c**) are the phase profile of the vortex beams with *l* = +1 and +2 at 0.9 THz, 1.0 THz and 1.1 THz, respectively. (**b**) and (**d**) are the OAM mode purity with *l* = +1 and +2 from 0.9 THz to 1.1 THz, respectively.

Moreover, the broadband operation of vortex beam generators has also been investigated at topological charge *l* = +1 and +2 as the demonstration. The near-field phase profile depicted in Figure 13a indicates that the characteristics of the spiral phase are almost identical. Moreover, the vortex beams with *l* = +1 simultaneously exist at a frequency of 0.9 THz, 1.0 THz and 1.1 THz. To quantitatively identify the difference of generated vortex beams at varied frequencies, the corresponding mode purity with *l* = +1 has been considered and depicted in Figure 13b. When the vortex beam with *l* = +1 is operating at a frequency between 0.90 THz to 1.10 THz by step of 0.05 THz, the mode purity is 94.0%, 93.6%, 94.5%, 92.3 and 93.9%, respectively. Similarly, the near-field phase profile and mode purity of the vortex beam with *l* = +2 from 0.9 THz to 1.1 THz are barely changed, which are depicted in Figure 13c,d. The negligible variations manifest the great stability of designed vortex beam generators in broadband operation.

## 4. Conclusions

In conclusion, a tri-layered metasurface composed of an arrow-type structure sandwiched by a pair of orthogonal gratings is proposed and investigated in detail. Specifically, the top and bottom gratings are employed as polarization selectors, while the middle layer performs the polarization convertor. The transmission coefficient of the polarization conversion metasurface is higher than 90% from 0.73 THz to 2.24 THz and the corresponding PCR is greater than 99.5%. In addition, the complete phase coverage of 0–2π at operation frequency can be obtained in the proposed metasurface by altering the geometric parameter. To demonstrate the concept of wavefront manipulation, the anomalous refraction, cylindrical focusing, point focusing and vortex beams with varied topological charges are investigated. The simulated results indicate that the anomalous refraction can be realized from 1.0 THz to 1.9 THz, and the focusing efficiency of cylindrical and point focusing metalens are 69.9% and 82.4%, respectively. Moreover, the mode purity is also considered to evaluate the efficiency of vortex beams and all the vortex beams have high mode purity greater than 90%. In addition, the broadband operation of point focusing and the vortex beam are also investigated, which exhibit stable performances in a certain frequency range. It is also worth pointing out that when the incident waves with *x*-polarization cross the metasurface along −*z* direction, the *y*-polarized transmitted waves with completely identical performances can be obtained. Therefore, the proposed metasurface can be functional in both +*z* and −*z* directions. The remarkable results show the proposed metasurface has great potential in prompting the efficient, broadband and compact systems for THz wireless communication.

## Data Availability

Data underlying the results presented in this paper are not publicly available at this time but may be obtained from the authors upon reasonable request.
